# Application and Algorithm of Ground-Penetrating Radar for Plant Root Detection: A Review

**DOI:** 10.3390/s20102836

**Published:** 2020-05-16

**Authors:** Hao Liang, Linyin Xing, Jianhui Lin

**Affiliations:** 1 School of Technology, Beijing Forestry University, Beijing 100083, China; lianghao@bjfu.edu.cn (H.L.); lyxing@bjfu.edu.cn (L.X.); 2 Beijing Laboratory of Urban and Rural Ecological Environment, Beijing 100083, China; 3 Research Center for Intelligent Forestry, Beijing 10083, China; 4 Key Lab of State Forestry Administration for Forestry Equipment and Automation, Beijing 10083, China

**Keywords:** root detection, machine learning, ground-penetrating radar

## Abstract

Attention to the natural environment is equivalent to observing the space in which we live. Plant roots, which are important organs of plants, require our close attention. The method of detecting root system without damaging plants has gradually become mainstream. At the same time, machine learning has been achieving good results in recent years; it has helped develop many tools to help us detect the underground environment of plants. Therefore, this article will introduce some existing content related to root detection technology and machine detection algorithms for root detection, proving that machine learning root detection technology has good recognition capabilities.

## 1. Introduction

Trees are a precious resource—as important as glaciers and oceans—and are the wealth of a country and society; thus, they are earth resources we must treasure. Trees play a very important role in maintaining natural ecosystems and regulating the climate and environment. However, due to development reasons, most countries have different levels of excessive deforestation, and it is urgent to protect forests and their ecological diversity. Of all the organs of a plant, the root is a vital organ. It is responsible for fixing plants, obtaining nutrients in the soil and improving soil composition. Therefore, it is very important to detect and evaluate the root system of existing plants in forests and other regions, and it has great development significance.

The roots are underground, but they account for 10–60% of the plant’s biomass. This feature limits our analysis and research on plant root systems. Destructive research methods are harmful to plants, which may cause them to fail to grow properly and even die. Therefore, non-destructive research methods have been generally accepted in recent years. The root system is analyzed and researched based on not destroying the original structure and ecological environment of the tree. Ground-penetrating radar (GPR) is a recognized research tool for protecting the ecology and can be used to locate buried objects, such as rocks, artifacts, public facilities and objects, as well as groundwater levels. In recent years, GPR has been used to detect and map plant root systems to determine root biomass and diameter. The reliability and practicability of the results of using GPR are widely accepted, and showing great potential. However, some research results have reached the opposite conclusion, which may present great difficulty for people when estimating the soil (a non-uniform medium). Therefore, a deeper understanding of the root system may improve the development and understanding of GPR in this field.

This article will introduce the basic GPR detection technology, as well as related technologies and machine learning supplements for further improvement and development of related technologies in the field. In recent years, the development of machine learning has shown an exponential trend. Among them, it has shown good results in the field of image processing and recognition segmentation. Based on previous research, we can apply these technologies to plant root recognition to find ways to improve performance. This article will analyze the basic technology of a back propagation neural network, as well as introduce the main achievements and shortcomings of various root detection technologies and image processing technologies, and hope to help the development of machine learning in the field of GPR in the future. In summary, the following will give an overview of GPR and deep learning applications in the following order:

- The GPR working principle and root detection processing technology.
- Important factors for estimating root biomass.
- Algorithms and machine learning achievements in the field of GPR.
- Prospects.

## 2. Ground-Penetrating Radar Principle and Detection Technology

As a non-destructive testing method, GPR is often used for non-destructive testing of shallow underground objects [1]. The working principle of GPR is based on electromagnetic field theory (EM), which is based on Maxwell’s equations. The validity of the measurement also depends on the reflection of the radar wave by the object, which is determined by the constitutive equation. Besides, the EM theory and the degree of physical properties of the material of the object are very important for analyzing the signal. To simplify the description, ground-penetrating radar is abbreviated as GPR in this paper.

### 2.1. GPR Theoretical Background

**Figure 1:**
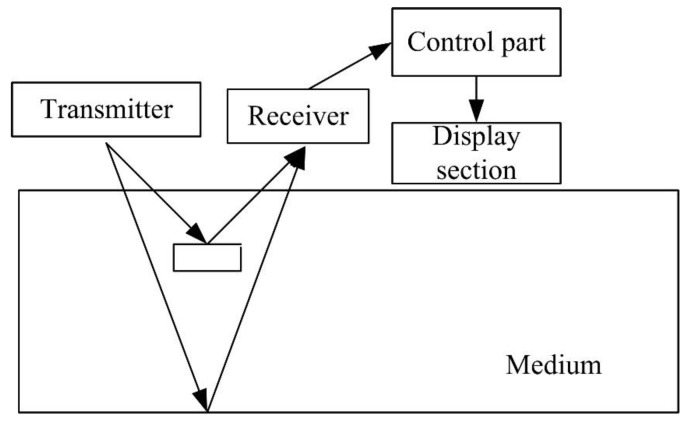
Work process of ground-penetrating radar (GPR).

The standard GPR system consists of the following parts: a control unit (pulse generator, computer and related software), antenna (transmitting antenna and receiving antenna) and display unit, as shown in Figure 1 [2,3]. During use, the GPR scanner sends transmitting EM energy pulses. These pulses are transmitted to the detection object in the form of waves. When these waves touch targets with different electromagnetic characteristics, they will be reflected. The reflected wave has then reflected the surface and is received by the receiving device. The remaining electromagnetic waves will continue to propagate through the medium until the energy drop to zero. The control unit receives the reflected electromagnetic waves and records, samples and filters the signals. Finally, it combines the signals onto a reflection trace (A-scan), records the time from the transmitted signal to the reflected signal perpendicular to the transmitting position and receives the amplitude of the signal. A-scan can provide real-time information below the target object.

The depth of the target object can be obtained by the propagation speed of the electromagnetic wave, as shown in Equation (1) below:

(1) $$D=V\times \frac{t}{2},$$

where D, t is the two-way propagation time. Similarly, we can also calculate the wave velocity:

(2) $$V=\frac{1}{\frac{\epsilon \mu }{2}(\sqrt{1+\left({\frac{\sigma }{\epsilon \omega }}^{2}\right)}+1},$$

where *μ* is the magnetic permeability; *σ* is the electrical conductivity; *ϵ* is the dielectric constant; and *ω* is the angular frequency (*ω* = 2*π*f, where f is the frequency). Besides, [4] estimated the propagation rate of low conductivity and non-magnetic materials.

**Figure 2:**
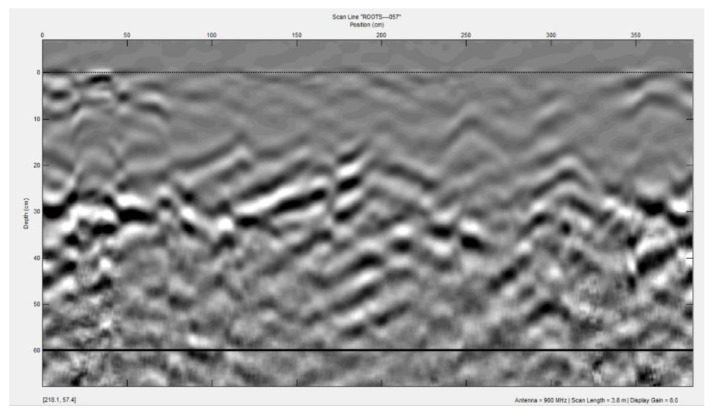
B-scan hyperbola image.

During the detection of an underground object, ground-penetrating radar moves along the detection line and generates EM pulses at manually specified intervals. After recording the reflected signal, the reflected EM trace can be integrated into the radar map to form a b-scan image to generate an underground 2D image (Figure 2). By using GPRMAX, you can simulate the establishment of B-scan images to simulate plants [5]. The introduction to GPRMAX will be presented later.

**Figure 3:**
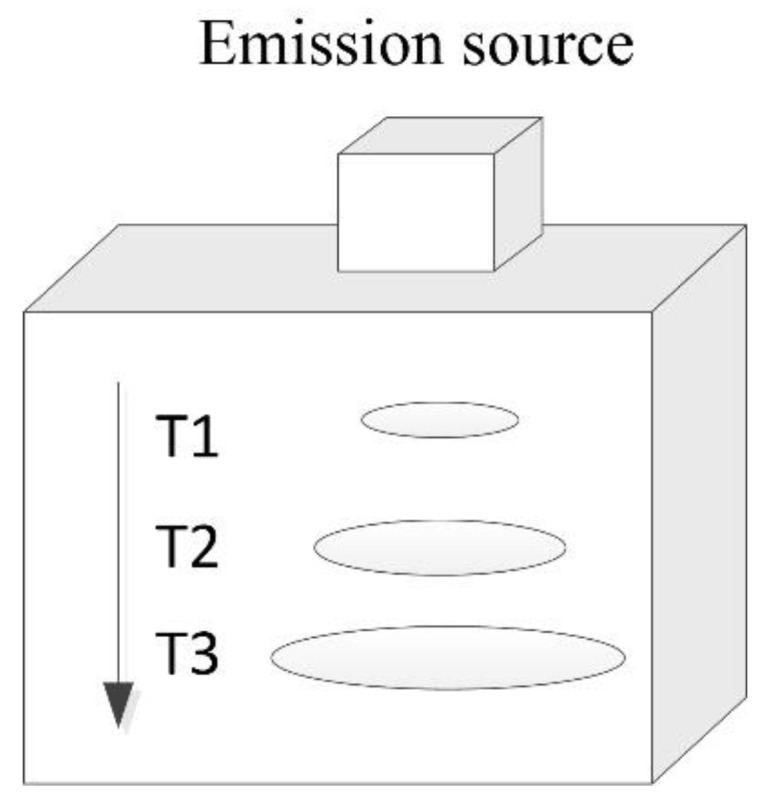
The electromagnetic wave waveform generated by the emission source.

The ground-penetrating radar emits electromagnetic waves that penetrate the ground in an ellipse. As the propagation depth increases, the radar wave will gradually expand, and the area scanned by the antenna wave will gradually increase (Figure 3). The coverage area can be expressed as follows:

(3) $$R=\frac{\alpha }{4}+\frac{D}{\sqrt{\varepsilon +1}},$$

R is the major axis radius in the radar wave scan, *α* is the center frequency wavelength of the radar energy; *D* is the depth from the ground to the reflecting surface; and *ε* is the average relative permittivity of the scanning material from the ground to the mirror depth (*D*).

**Figure 4:**
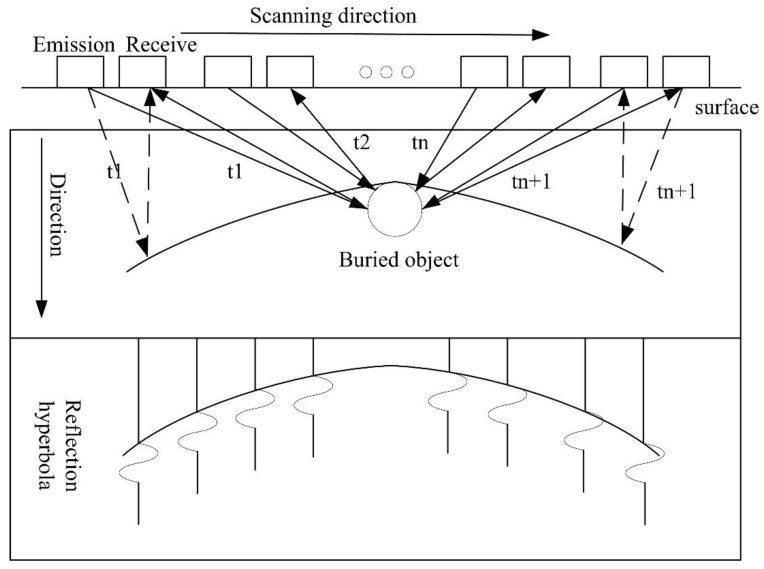
Schematic diagram of GPR reflection hyperbola generation.

Based on the reflection characteristics of the electromagnetic waves, radar energy will be reflected before and after the antenna is placed over a buried object. When the transmitting antenna moves closer to the target, the propagation time recorded at the receiving end decreases, and when the antenna moves away from the target, the same phenomenon repeats, resulting in a hyperbola of reflection, whose apex indicates the exact location of the buried object (Figure 4).

GPR resolution and its ability to distinguish between two closely spaced targets and the smallest detectable size are negatively correlated with the coverage area. The GPR detection resolution depends on the antenna frequency, the EM characteristics of the medium, and the penetration depth [6]. Therefore, in the use of GPR, the frequency, the type of antenna and its polarization depend on many factors, such as the size and shape of the target and the transmission characteristics of the medium under investigation, as well as the surface features [7]. The resolution of the GPR in detecting adjacent targets and detecting the smallest object size is inversely proportional to the coverage area. The detection resolution of a GPR depends on the antenna frequency, the EM characteristics of the propagation medium and the penetration depth [8]. Therefore, using ground-penetrating radar for detecting objects will depend on the shape and size of the detection surface features and the characteristics of the transmission medium to select a different model GPR, such as the type of signal frequency and antenna.

Over time, the long and short axes of the ellipse generated by the electromagnetic wave will continue to increase.

With the development of GPR data processing technology and visualization technology, it is already possible to build underground 3D images (also known as C-scan). C-scan can provide radar wave amplitude maps at specific times or depths. On this basis, the requirements for signal processing technology will increase, and better processing technology is needed to provide researchers with more intuitive underground 3D reconstruction graphics [6].

### 2.2. Root Detection Technology of Ground-Penetrating Radar

Ten years ago, GPR was widely used in cities [9], archeology [10] and other aspects, and the root system of plants is generally considered as an impact object that interferes with research. But in the past decade, GPR has gradually been adopted by scholars to draw plant root images, because it is protective and can be repeatedly measured. The first application of ground-penetrating radar to the detection of plant roots can be traced back to 1999. In this study twenty years ago, a GPR system with a center frequency of 450 MHz was used to draw a 50-year-old rough root map of an oak tree, as well as measured them in two directions of 0.25 m, in a 6 m × 6 m square × 0.25 m contour grid with an interval of 0.05 m. After data processing, the depth correlation of the GPR was applied to analyze the root system of the big oak tree in detail to make a three-dimensional underground image. After the research, the root system was dug and photographed, and the length and diameter of the roots were measured to verify the radar data. Researchers have confirmed that the resolution of the GPR system is sufficient to distinguish roots with a diameter of 3 to 4 cm. Compared with the measured diameter of the excavated root, the diameter of the root detected by the GPR system has an error of 1–2 cm. GPR determines the length of a single root, from the stem to the smallest detectable width, with an error range of about 0.2 to 0.3 dm. Applying higher frequencies and shorter measurement intervals, this method improves resolution and accuracy to less than 1 cm. In summary, this study demonstrates that GPR has been successfully tested in relatively homogeneous forest and woodland environments. A few years later, the study’s conclusions were criticized because the 3D view of the study was drawn manually from the GPR radar chart, but did not provide specific information on how it was done. If the map is redrawn based on researcher experience, the accuracy of the technology can be misjudged.

From this point of view, the three-dimensional reconstruction of plant roots has been researched [11,12]; however, the results are not satisfactory, which shows that there is still a wide space for development. The biggest difficulty in using GPR to map plant root systems is the resolution of the GPR. When the radar scan is in an area with dense root systems, a single root cannot be identified because there is only one huge parabola. In this light, some research is aimed at limiting the detection of tree roots and the determination of root diameters by ground-penetrating radar under optimal conditions. The minimum resolution of GPR is still evolving. Tests so far under controlled conditions have confirmed that fine roots with a diameter of 0.5 cm or less can be detected [13].

## 3. Important Factors for Root Detection

### 3.1. Relative Angle

**Figure 5:**
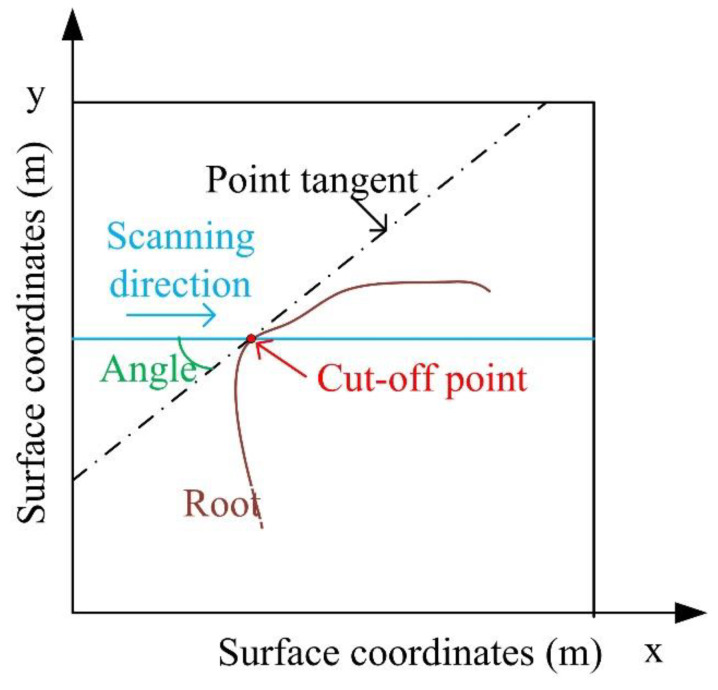
Definition of the included angle.

In the process of using radar forward modeling tools to simulate the generation of a large amount of GPR signal data, the parameters of various biomass in the root system, including direction, dielectric constant, etc., need to be relatively correct. Several studies have shown that the relative angle between the scanning direction of the GPR and the root direction affects the amplitude of the reflected waveform, and then affects the judgment of the root diameter [5,14,15]. As shown in Figure 5, when viewed from the plan view, the GPR scanning direction (blue line) is at the intersection (red point), intersecting the root branch (brown curve). The tangent direction at this intersection is defined as the root direction and is represented by a dashed line. The horizontal crossing angle between the measurement line and the root direction indicates the relative position between the root and the measurement line. Because of the direction of the root changes along the curved root branch, the crossing angles formed at different intersections change. The experimental data show that when the scanning direction is perpendicular to the rhizome direction, the result of forwarding modeling is very close to the actual value. However, when the intersection angles are (45°, 60°), (60°, 75°), (105°, 120°) and (120°, 135°), the estimated result is not accurate enough.

### 3.2. Dielectric Constant of Soil

In soil exploration, the dielectric constant determines the main characteristics of the soil and it is a very important factor in ground-penetrating radar detection. Studies have shown that the factors that affect the dielectric constant of unsaturated soils are salt, moisture, organic salts and compaction [16]. Common dielectric constant measurement methods include the transmission line impedance conversion method, reflected light polarization method and four-terminal measurement method, which is not repeated here. Besides, we can also establish the corresponding dielectric constant model based on the obtained relevant data. Reference [17] gives the common formula of the soil dielectric constant:

(4) $$\varepsilon_{s}=3.03+9.3\theta_{v}+146.0\theta_{v}^{2}-76.7\theta_{v}^{3},$$

where $\varepsilon_{s}$ is the soil dielectric constant, and $\theta_{v}$ is the soil moisture content.

### 3.3. Electrical Conductivity of Soil

The main factors affecting soil electrical conductivity are the soluble salt, clay content and water content, and the effect of water content is the most significant [17,18]. Under normal circumstances, the effect of the soluble salt and clay content is negligible, so the soil conductivity can be approximated by the following formula [19,20]:

(5) $$\sigma =4.504e^{8.2635\theta },$$

where σ is the electrical conductivity of the soil, and *θ* is the volumetric water content.

### 3.4. Dielectric Constant of Plant Roots

There are three commonly used root dielectric constant models: the Power Law Model, MG Model and PS Model. These models are based on the assumption of the mixed theory that fixed-shaped fillers are dispersed in an environmental state, namely the body and the matrix. In wood biomass, the air is the main body, while a solid solution and water are the filler or matrix [17]. Ana Paz et al. proposed a formula that can calculate the dielectric constant of the root system based on the model designed by their predecessors:

(6) $$\varepsilon_{m}^{\prime \beta }=\theta_{fw}\times \varepsilon_{fw}^{\prime \beta }+\theta_{a}\times \varepsilon_{a}^{\prime \beta }+\theta_{s}\times \varepsilon_{{}_{s}}^{\prime \beta },$$

where *θ* represents the volume of the corresponding plant root system; *m* represents the mixture; *fw* represents free water; *a* represents air; and *s* represents a solid solution.

### 3.5. Electrical Conductivity of Plant Roots

For the root conductivity with water content below the fiber saturation point (FSP), Straube established a numerical relationship in 2002 and proposed an approximate calculation formula:

(7) $$Log_{10}WC=2.99-2.113(log_{10}log_{10}\frac{1}{EC}),$$

where, among them, *WC* and *EC* are the water content and conductivity in wood materials.

## 4. Application of Machine Learning in GPR

### 4.1. BP Neural Network

The BP neural network is widely used in various root recognition technologies [21], such as the Hough transform [22] and C3 algorithm (Column Connection Clustering) [23]. The back-propagation learning algorithm can help us identify hyperbolic features and classify them. To better understand how neural networks help us identify the characteristics of GPR signals, neural networks will be introduced in detail.

**Figure 6:**
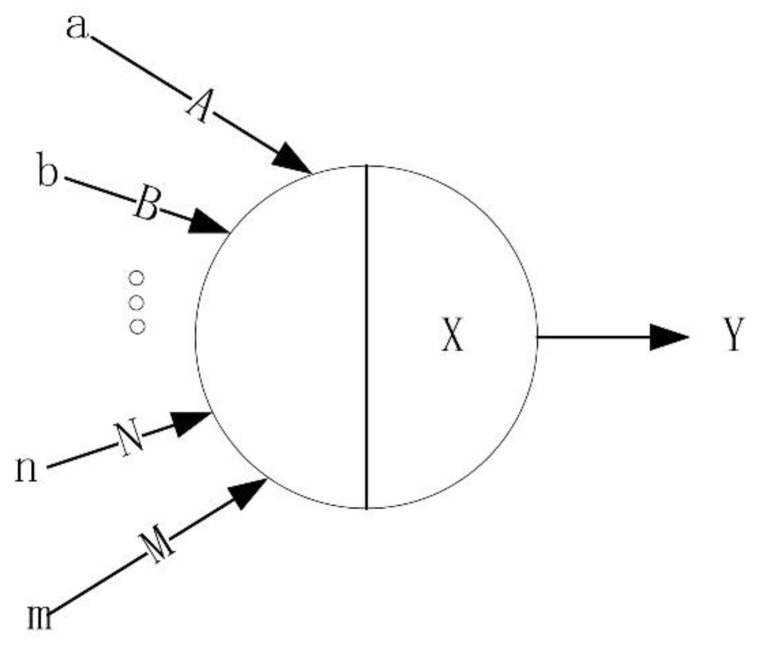
Single neuron model.

Neural networks are widely parallel interconnected networks of adaptively simple units whose organization is capable of simulating the interactive response of biological nervous systems to real-world objects [24]. To better fit the input data, the BP neural network will automatically reduce the deviation continuously [25]. The following Figure 6 is a simple neuron model [26]:

In Figure 6, a, b ... n, m represents the input of the neuron, and A, B ... N, M represents the weight of each input in the previous layer. X represents the threshold of neurons in this layer, and Y is the output of the neurons. Let a, b ... n, m represent *x*_1_, *x*_2_ … *x_m_*, A, B ... N, M represent $\omega_{1},\omega_{2}\ldots \omega_{n}$, and the formula for calculating Y is

(8) $$Y=f\left(\overset{m}{\underset{i=1}{\sum }}x_{i}\omega_{i}-\theta \right).$$

Each neuron is connected through a weighted input. The sum of the input weights inside the neuron is compared with the neuron threshold. Finally, it is output through the activation function to enter the next neuron as the next input.

#### 4.1.1. Activation Function of the Neural Network

Two commonly used activation functions are the sigmod and relu functions. The function formula is as follows:

(9) $$sigmod\left(z\right)=\frac{1}{1+e^{-z}},$$

(10) $$relu\left(z\right)=\left\{\begin{array}{l} z,z>0\\ 0,z\leq 0 \end{array}\right\}.$$

The significance of introducing the activation function is to solve the linear inseparable problem. Neural networks without activation functions are purely linear networks. Adding various forms of activation functions is equivalent to introducing non-linearity. To speed up the calculation and reduce the trend of gradient descent, a relu function is generally introduced in the middle of the neural network.

#### 4.1.2. Neural Networks and Cost Functions

In a simple three-layer neural network, let us suppose one layer is the input layer, the second layer is the hidden layer and the third layer is the output layer. Let $\omega_{jk}^{l}$ represent the weight matrix of the *k*th neuron in the (*l*-1) layer pointing to the *j*th neuron in the *l*th layer; $b_{j}^{l}$ represent the offset of the *j*th neuron in the lth layer; $z_{j}^{l}$ represent the output of the *j*th neuron in the first layer; and $a_{j}^{l}$ represent the output of the *j*th neuron in the lth layer after the activation function. Then, the expression of the forward propagation matrix for *m* sample inputs is

(11) $$a^{\left(l\right)}=\sigma \left(\omega^{\left(l\right)}a^{\left(l-1\right)}+b^{\left(l\right)}\right),$$

where $a^{\left(l-1\right)}$ represents an n * m matrix, *n* is the number of neurons in layer *l*-1 and *m* is the number of input samples.

As for the cost function of the neural network, the main idea is to make the difference between the actual value and the fitted nonlinear function to get the difference between the fitted function and the actual data. In order not to be affected by individual extreme data, half of the variance is generally adopted to reduce the impact of individual data. The following formula gives only one kind of cost function expression; in addition, there are multiple cost function expressions, such as the logarithm, which will not be repeated here. $h_{\theta }\left(x\right)$ represents the linear output through the activation function.

(12) $$J\left(\theta_{0},\theta_{1}\right)=\frac{1}{2m}\overset{m}{\underset{i=1}{\sum }}{\left(h_{\theta }\left(x^{\left(i\right)}\right)-y^{\left(i\right)}\right)}^{2}.$$

#### 4.1.3. Backpropagation Algorithm

The core idea of the BP backpropagation algorithm is to reduce the error between the output layer and the expectation by adjusting the network parameters. The core of the backpropagation algorithm follows the following four formulas:

Output layer error expression, where $\delta_{j}^{\left(L\right)}$ is the error generated by the *j*th neuron output in the Lth layer; the right side of the equation shows the partial derivative of the output layer loss function for the *j*th neuron in the *L* layer, and the derivative of activation function of *j* neurons linear output.

(13) $$\delta_{j}^{\left(L\right)}=\frac{\partial L}{\partial a_{j}^{\left(l\right)}}\sigma^{\prime }\left(z_{j}^{\left(l\right)}\right).$$

Hidden layer error expression, where $w_{kj}^{\left(l+1\right)}$ presents the weight of the *j*th neuron in the *l*th layer to the *k*th neuron in the *l* + 1th layer.

(14) $$\delta_{j}^{\left(L\right)}=\underset{k}{\sum }w_{kj}^{\left(l+1\right)}\delta_{k}^{\left(l+1\right)}\sigma^{\prime }\left(z_{j}^{\left(l\right)}\right).$$

The above formula is the gradient of parameter change and parameter update expressions; the expression indicates that the loss function modifies the gradient of weights and offsets through varying errors. Besides, according to the minimum gradient method, the parameter should change in the direction that the derivative becomes smaller, so it takes a negative sign here. According to the minimum gradient method, the parameter should also change in the direction that the derivative becomes smaller, so it should take a negative sign here. In the research process of improving the BP algorithm, some scholars proposed to increase the momentum direction, adjust the adaptive rate, RMS and other techniques to eliminate the shortcomings of the BP algorithm, such as the inability to obtain the optimal solution, too many iterations and forgetting the old samples. By using the improved algorithm, we think it allows us to better use the BP algorithm for the root recognition image recognition process.

(15) $$\frac{\partial L}{\partial b_{j}^{\left(l\right)}}=\delta_{j}^{\left(l\right)},$$

(16) $$\frac{\partial L}{\partial \omega_{jk}^{\left(l\right)}}=a_{k}^{\left(l-1\right)}\delta_{j}^{\left(l\right)},$$

(17) $$b_{j}^{\left(l\right)}=b_{j}^{\left(l\right)}-\alpha \frac{\partial L}{\partial b_{j}^{\left(l\right)}},$$

(18) $$\omega_{jk}^{\left(l\right)}=\omega_{jk}^{\left(l\right)}-\alpha \frac{\partial L}{\partial \omega_{jk}^{\left(l\right)}}.$$

### 4.2. Root Detection Algorithm

#### 4.2.1. Genetic Algorithm (GA)

The genetic algorithm is well known in optimization algorithms for its stability and efficiency. In each iteration of the algorithm, the genetic algorithm retains many candidate solutions. In addition, gradient descent is not used, but direct optimization based on the objective function. Genetic algorithms have been widely used successfully, including, for example, biology, chemistry, control systems, electromagnetics and image processing [27,28].

GA uses the “chromosome” as a model to search for the optimal solution by simulating the natural evolution process. From one generation to the next, the algorithm is improved through a genetic mechanism, by using deterministic and non-deterministic genetic operators. The most common form of GA consists of the following steps. First, an initial chromosome population is randomly generated. The optimality of each chromosome is then evaluated according to a predetermined fitting function, representing the objective function under consideration. This adaptive assessment step will maintain the best chromosomes and eliminate the worst chromosomes by using appropriate selection rules based on the principle of higher fitness and a higher chance of being selected. Once the selection process is complete, the next step is to breed the population. This is done by genetic operators, such as crossover and mutation. The crossover algorithm is a deterministic operator similar to biological exchange, and its purpose is to combine two chromosomes (parents) selected according to the roulette rotation method to generate new chromosomes (offspring). The basic idea of this algorithm is to produce offspring. If offspring inherit the best genes of their parents, they may perform better than their parents. The mutation is a random operation, similar to a biological mutation, which randomly changes one or more genes of the chromosome in question. This operator can be used to prevent the population from falling into a local optimum. Iterate the entire process until a user-defined convergence criterion or a maximum number of iterations is reached. Through the genetic algorithm, the B-scan hyperbola can be successfully identified, and the types of underground buried substances can be further identified through processing, such as windowing, energy matching and normalization [29].

Through the description of the genetic algorithm, it has the following advantages: it can find the global optimal solution of the optimization problem, the optimization result has nothing to do with the initial conditions and it is widely used. But at the same time, it also has the disadvantages of a slow convergence, more control variables and a poor local search ability. In view of the characteristics of the genetic algorithm, it is widely used in combinatorial optimization problems. Therefore, we think the algorithm can be used in the automatic control of ground-penetrating radar.

#### 4.2.2. Expectation Maximum Algorithm

In statistical calculations, the expectation maximum algorithm is an algorithm that looks for the maximum likelihood estimation or the maximum posterior estimation of parameters in a probabilistic model, where the probability model depends on an unobservable hidden variable (latent variable). The biggest expectations are often used in the field of data clustering for machine learning and computer vision. The expectation maximum algorithm is calculated alternately through two steps. The first step is to calculate the expectation (E) and use the existing estimated value of the hidden variable to calculate its maximum likelihood estimate. The second step is to maximize (M); the maximum likelihood value obtained in step E is calculated to calculate the value of the parameter. The parameter estimates found at step M are used in the next step E calculations, and the process continues alternately.

When determining the hyperbolic model, we need to determine the number of the hyperbola. The number of a hyperbola can be well determined through the Bayesian information criterion, and the position and shape of the hyperbola can be determined by using the expectation maximum algorithm. This type of method is very effective for multi-channel GPR systems and can generate a large amount of data on various tests. Therefore, research in this area will also likely play an important role in GPR and related industries (such as utility inspection, infrastructure and transportation industries) [29]. Expectation maximum algorithms are often used in the field of data clustering in machine learning and computer vision. It is not suitable for large-scale data sets and high-dimensional data, but the calculation structure is stable and accurate. The EM algorithm can iterate according to the existing data to make it converge to the optimal solution. Therefore, it can be used in the image recognition process of root detection [30,31].

#### 4.2.3. C3 Column Link Clustering Algorithm

The column link clustering algorithm is a root detection and recognition method based on neural networks. The C3 technology used in this method is based on the connection relationship between the pixels in the image. Not only can the branches of the cross hyperbola be separated and identified, but also the input is small, and it can achieve good results with fewer data training. In the artificially synthesized B-scan image, there is a full recognition accuracy for 52 sets of data, and few hyperbola intersections are misrecognized. In the real scan processing results, the number of hyperbola identified will increase compared to the noise of the synthesized data.

**Figure 7:**
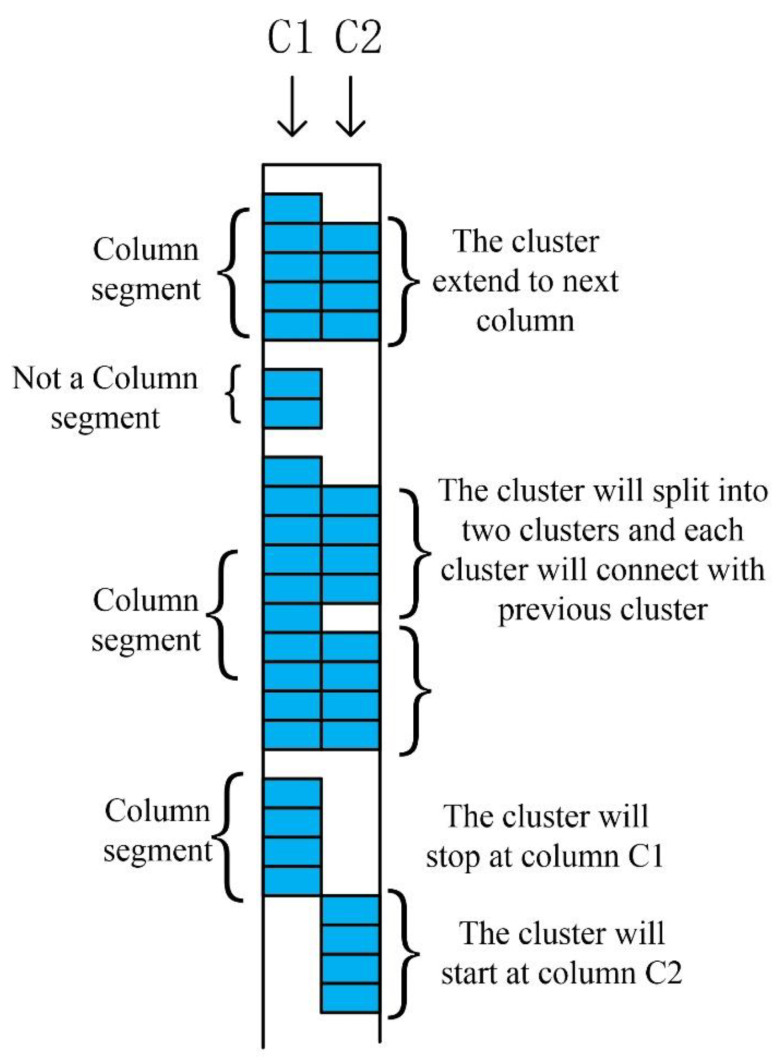
Three situations generated during scanning.

In the column link clustering algorithm, the pre-processed image is subjected to a column scan and clustered according to the vertical link between the pixels to divide into multiple column clusters. Then, each column cluster is identified according to the radar scanning direction, and the parts connected to the column cluster are integrated into a cluster. The three situations generated at this time are shown in Figure 7.

First, we define a cluster of pixels with more than four nodes. In the first cluster of column C1, since there are four connection points with C2, they are a cluster. In the second cluster of column C1, because there are only two pixels, it is excluded. In the third cluster of C1, because there are discontinuities between the eight pixels connected to C2, the third cluster of C1 and the second cluster of C2 are merged into the a1 cluster, and the third cluster of C1 and the third cluster of C2 are merged into the b1 cluster. In the fourth cluster of C1 and the fourth cluster of C2, since they are not connected, they are regarded as a single cluster. The rest of the image works on the same principle; this way it links the entire image. If the situation shown in Figure 7 occurs, the derivative of the intersection p is judged. If it is a hyperbola with a downward opening, the first derivative of p should be 0 and the second derivative is negative. After the method is run, the image is automatically recognized by combining it with the neural network [23]. Finally, the detection rate between several methods is obtained, as shown in Table 1. Through the introduction of the C3 algorithm, we can try to improve the number of clusters in the future to adapt to different pictures to achieve the optimal solution, so as to better perform root recognition. The comparison in the table fully demonstrates its high level of accuracy in GPR identification and is one of the effective tools that can continue to provide assistance for root detection in the future.

#### 4.2.4. Faster R-CNN

**Figure 8:**
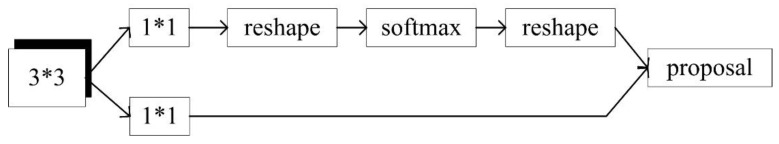
The Region Proposal Network.

Faster R-CNN is mainly composed of four parts: Conv layers, Region Proposal Networks, Roi Pooling and Classification. As a ground-penetrating radar simulation software, GPRMAX can well fit the construction of various underground environments, providing us with the possibility of obtaining a large amount of forwarding data. As shown in Figure 8, it can be seen that the Region Proposal Network is actually divided into two lines. The upper one uses the softmax classification anchors to obtain the positive and negative classifications. The next one is used to calculate the bounding box regression offset for the anchors to obtain accurate proposals. The final Proposal layer is responsible for synthesizing positive anchors and corresponding bounding box regression offsets to obtain proposals, while excluding proposals that are too small and beyond the boundary. In fact, when the entire network reaches the Proposal layer, it has completed the function equivalent to target positioning. The RoI Pooling layer is responsible for collecting proposals and calculating the proposal feature maps, which are then sent to the subsequent network. The Classification part uses the already obtained proposal feature maps, calculates the specific category of each proposal through the full connect layer and softmax, and outputs the cls prob probability vector; at the same time, it again uses the bounding box regression to obtain the position offset of each proposal for regression; a more accurate target detection frame. In [32], the location and detection of mines were realized by using Faster R-CNN, and some scholars used this method to realize the classification function of underground targets [33]. The relationship between the depth and the image pixels, as well as the width of the antenna beam, is crucial for the implementation of this approach.

Compared to RCNN, Faster-RCNN no longer separately extracts features, which improves speed. However, it takes a long time to extract candidate frames using selective search. Although Faster-Rcnn still cannot achieve the real-time detection target, its detection accuracy and speed are very high, which is very suitable for the research and analysis process of plant roots rather than commercial use.

### 4.3. Application Status

Through the introduction of the neural network, we can input the GPR image information into the neural network, extract the feature values in the image to achieve the goal of identifying hyperbola and further features. Because machine learning involves a large amount of data analysis, the field generally cannot obtain a large amount of image information. As a ground-penetrating radar simulation software, GPRMAX can well fit the construction of various underground environments, providing us with the possibility of obtaining a large amount of forwarding data.

#### 4.3.1. Forward Simulation Software GPRMAX

GprMaxV2.0 was originally from the doctoral dissertation of Dr. Antonis Giannopoulosd of the University of Edinburgh, as open-source software, and is constantly updated and developed. The finite-difference time-domain method (FDTD) is the basic theoretical basis of the GprMax simulation, and it is a commonly used method in the field of electromagnetic field calculation. The finite-difference time-domain method divides the research space into a certain grid form and then uses the finite-difference equations to approximate the Maxwell equations in the time domain. As an open-source ground-penetrating radar simulation software, the main functions of GprMax includes a simple command interface, modeling of simple media models, modeling of complex shape targets and simulation of unbounded space using absorbing boundary conditions. GprMax3D can simulate GPR antennas and even introduce its feed transmission lines into the model. GprMax2D is mainly used for GPR “signature” simulation, while GprMax3D is used for more detailed and realistic simulation, especially when compared with real GPR data. Both GprMax2D and 3D programs use simple ASCII (text) files to define the parameters of the model. The software has a very detailed user manual in which you can find detailed information on the functions of the program. For the processing of root scanning, we use GprMax3D for modeling and simulation, so as to obtain a large number of approximate real data to approximate the scanned image generated by GPR in the actual measurement. The simplest medium is a homogeneous, linear and isotropic medium, and its constitutive relationship is j=δE, D=εE and B=μH. In the formula, δ is the electrical conductivity, ε is the dielectric constant, μ is the magnetic permeability and all are scalar constants, as well as parameters of the electrical properties of the reaction medium; *J* is the current density, *D* is the electric displacement vector, *B* is the magnetic induction strength, *E* is the electric field strength, and *H* is the magnetic field strength. GprMax software is used for forward modeling of ground-penetrating radar data, which has been widely used at home and abroad [34].

#### 4.3.2. Application Section

**Figure 9:**
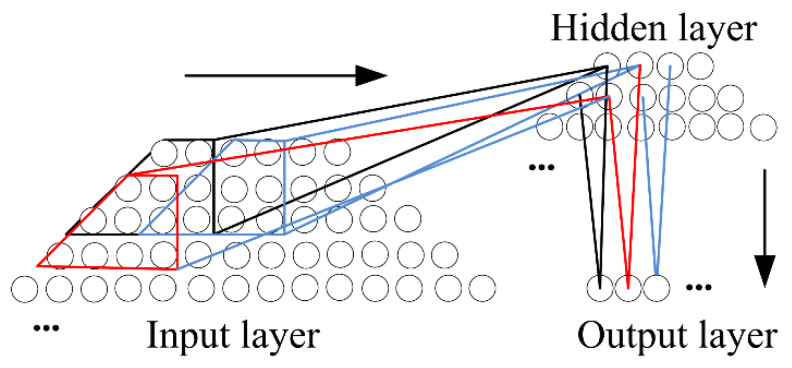
The feature extraction process of a neural network.

As shown in Figure 9, the continuous scanning of hyperbolic images is discretized by deep learning. A general backpropagation algorithm is used. The partial set of pixels is used as the input of the next hidden layer, which reduces the amount of computer processing and increases sensitivity to hyperbola. At the same time, the hyperbola is also split into two parts. Due to its symmetrical structure, the analysis side can increase the calculation speed by almost half [35].

**Figure 10:**
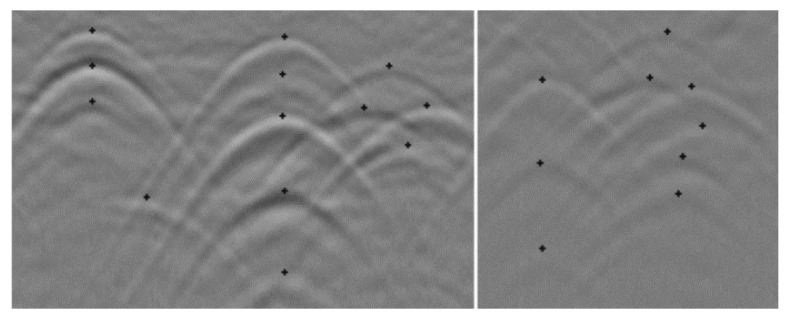
Reliable classification results in case of interfering hyperbolas.

Due to the complexity of some terrains, for many types of underground environments, such as cables, the obtained B-scan will recognize many similar hyperbolas. At this time, it is difficult to determine the recognition accuracy and the exclusion of environmental influence factors. Decreasing the recognition accuracy may cause the plant part to fail to recognize, and the high accuracy may cause the recognition to be inaccurate. In addition, the images obtained through forward modeling are still different from the actual environment, and the results obtained will also be partially biased. In [33], the training set used consists of nearly 1000 half trapezoids taken from over 100 radargrams. Approximately 40% of them contain hyperbolas from a large variety of objects in different soils and depths; the other 60% are a collection of background examples, non-hyperbolic reflections of any kind and geological structures. Due to the system’s sensitivity, it can cope very well with interfering or incomplete hyperbolas, which was one of the main goals of this work. Figure 10 shows two examples of such situations.

**Figure 11:**
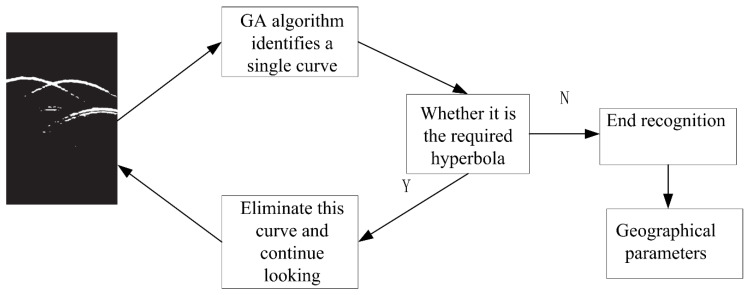
The root detection process of the genetic algorithm.

Figure 11 is a flow chart of curve recognition using the genetic algorithm. After several cycles, a large number of major hyperbola will be identified, and the last recognition is less than the threshold, which proves that there is no data to be identified in the image, and the remaining is noise and unwanted data. Then, the hyperbola obtained from the recognition is transformed into corresponding geographic horizontal and vertical coordinates, and the function of root detection can be realized. Reference [27] uses three materials (quartz stone, metal and air) and three different shapes (a circular section, square section and uniform layer) to evaluate the sensitivity to multi-object interference. In other words, the article verifies whether the classification accuracy depends on the distance between adjacent buried objects. They change the parameter d according to four different values: $\lambda_{0}$, $\lambda_{0}/2$, $\lambda_{0}/4$ and $\lambda_{0}/8$. The orientation angle of the two objects is randomly selected at intervals of (-π/2, π/2), and get the best performance when there is only one object on the ground. In addition, the closer the adjacent objects, the lower the object discrimination ability. But even if the objects are very close to each other (≥0.125λ0, i.e., about 9 cm), the classification accuracy is still satisfactory. The test results are shown in Table 2. From the 40 GPR scenarios, 25 (62%) were correctly processed in terms of detection, i.e., all the buried objects present in the corresponding image were correctly detected.

**Figure 12:**
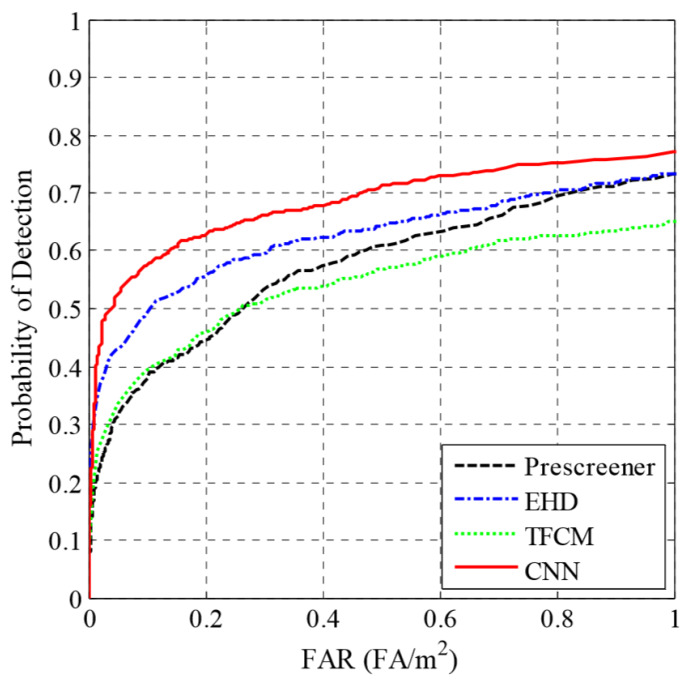
ROC curves summarizing the performance of our anomaly detection and discrimination algorithms.

In addition, there is a technique for preprocessing the B-scan using convolutional neural networks. After reducing the size of the picture to 49 * 49, learning the classification algorithm has also achieved good results [36], and a comparison of detection accuracy of several machine learning methods was obtained (Figure 12). Besides, there are new methods that developed a novel near-real-time, forward modeling approach for GPR that is based on a machine learning (ML) architecture. The ML framework uses an innovative training method that combines a predictive principal component analysis technique, a detailed model of the GPR transducer and a large data set of modeled GPR responses from our FDTD simulation software, and the accuracy of the ML-based forward model is a significant step toward commercially viable applications of FWI of GPR [37,38,39].

## 5. Summary and Outlook

This article introduces GPR (this article provides some information about GPR), including the working principle and calculation formula. Then, we briefly commented on some algorithms, and introduced several commonly used algorithms in root detection, combined with the neural network introduced later to achieve better results. Based on the various algorithms and data results introduced above, the technology of automatically identifying underground buried objects through machine learning has proved to be feasible and of great significance. After nearly two decades of development, GPR has made great progress in algorithms and optimization, but still faces difficulties. Through the genetic algorithm, backpropagation neural network and EM clustering algorithm introduced in this article, the continuous improvement by scholars in recent years shows that there is still much room for improvement in optimization. The optimization of machine learning algorithms is constantly evolving, and we can use the results of recent algorithm research for this research. Despite its good image processing and recognition technology, machine learning is rarely used in GPR. By using GPRMAX simulation software to generate a large amount of data, importing machine learning data sets for training can realize the recognition function of underground objects. The combination of machine learning and ground-penetrating radar can be used not only for root cause detection, but also for detecting any objects in the ground without having to model the objects separately. According to the working principle of GPR, we can also add machine learning technology in all aspects of scanning, recognition and display in the future. Finally, this article aims to focus on the application of machine learning. Many of the same excellent processing techniques are not repeated here, but this does not mean that they are not important.

In summary, we think machine learning methods can be used in the following aspects of ground-penetrating radar:

(A) Root detection and identification. After obtaining the B-scan map obtained by scanning the ground system of the ground-penetrating radar, accurate identification methods (such as Faster-RCNN) are used to conduct research for scientific personnel, and rapid identification methods can also be used to provide potential possibilities for commercialization.
(B) Commercial use. In view of the fact that some of the algorithms mentioned above have the characteristics of rapid convergence recognition (such as the C3 algorithm), they can be used for real-time measurement to achieve commercial use.
(C) Use machine learning techniques to improve GPR functions. Due to the limitations of the GPR output image, we can improve the picture quality from the source, so as to better carry out the subsequent research on the data.

Since 1999, the inversion of the underground environment is moving in different directions and using different methods. With the development of the times, we believe that machine learning can help better in the future.

## Figures and Tables

**Table 1:** Comparison of the average detection rates and fitting rates among different methods.

Method	Recall	Precision	F-Measure
Detection rates of [29]	0.724	0.347	0.474
Fitting rates of [29]	0.418	0.091	0.149
Fitting rates of improve C3 algorithm	0.704	0.708	0.702

**Table 2:** Genetic algorithm performances at the scenarios level.

Simulated Scenarios	Correctly Detected Scenarios	Correctly Detected and Recognized Scenarios
40	25 (62%)	18 (45%)
